# Dr. Alfred Ashby Shillitoe

**Published:** 1912-07-27

**Authors:** 


### Obituary.

Dr. Alfred Ashby Shillitoe, of Sydenham Hill, has died at Bournemouth at the age of forty-nine. Before joining his father, Dr. Burton Shillitoe, a well-known practitioner in South London, Dr. A. A. Shillitoe held several resident hospital appointments. He was house surgeon at the Seamen's Hospital, Royal Albert and Victoria Docks; at the General Hospital, Nottingham; and at the General Infirmary, Hertford. Dr. Shillitoe studied at St. Bartholomew's Hospital, and after taking the Conjoint Diploma in 1892 obtained the M.B., B.C. degree at the University of Cambridge.

